# Supplementary material for: SOX2 Expression Is an Independent Predictor of Oral Cancer Progression
Source: J Clin Med. 2019 Oct 21;8(10):1744. doi: 10.3390/jcm8101744 (PMC6832966; doi:10.3390/jcm8101744)
Supplement: Supplementary file 1 [file jcm-08-01744-s001.pdf]

Supplementary Table S1. Clinical and pathological characteristics of the 55 patients with oral epithelial dysplasia.

**Variable**	**Number (%)**
Age (years) (mean ±SD; median; range)	62.61 ± 12.56; 60; 39 - 83
Gender	
Men	26 (47)
Women	29 (53)
Tobacco use	
Smoker	10 (18)
Non-smoker	21 (38)
Unknown	24 (44)
Alcohol use	
Drinker	4 (7)
Non-drinker	27 (49)
Unknown	24 (44)
Location	
Tongue	20 (36)
Floor of the mouth	2 (4)
Gum	14 (25)
Buccal	13 (24)
Palate	2 (4)
Other sites within the oral cavity	4 (7)
Epithelial dysplasia	
Mild	42 (76)
Moderate	6 (11)
Severe	7 (13)
Clinical status at the end of the follow-up	
Progression to carcinoma	12 (22)
Remained as epithelial dysplasia	43 (78)

Supplementary Table S2. Clinical and pathological characteristics of the 125 patients with OSCC selected for study.

Variable	Number (%)
Age (years) (mean ±SD; median; range)	58.69 ± 14.34; 57; 28 - 91
Gender	
Men	82 (66)
Women	43 (34)
Tobacco use	
Smoker	84 (67)
Non-smoker	41 (33)
Alcohol use	
Drinker	69 (55)
Non-drinker	56 (45)
Location of oral squamous oral cell carcinoma	
Tongue	51 (40)
Floor of the mouth	37 (30)
Other sites within the oral cavity	37 (30)
Tumor status	
pT1	27 (22)
pT2	54 (43)
pT3	16 (13)
pT4	28 (22)
Nodal status	
pN0	76 (61)
pN1	25 (20)
pN2	24 (19)
Clinical stage	
Stage I	20 (16)
Stage II	32 (26)
Stage III	26 (20)
Stage IV	47 (38)
G status	
G1	80 (64)
G2	41 (33)
G3	4 (3)
Second primary carcinoma	
No	106 (85)
Yes	19 (15)
Local recurrence	
No	71 (57)
Yes	54 (43)
Clinical status at the end of the follow-up	
Live and without recurrence	53 (42)

Dead of index cancer	53 (42)
Lost or died of other causes (censored)	19 (16)

Supplementary Table S3. Relationship between the clinicopathological variables and SOX2 expression in OSCC patients.

Variable	*Number of cases*	Positive SOX2 expression (%)	*P*
Gender			
Men	80	32 (40)	0.87
Women	41	17 (41)	
Tobacco use			
Smoker	81	34 (42)	0.63
Non-smoker	40	15 (37)	
Alcohol use			
Drinker	66	29 (44)	0.39
Non-drinker	55	20 (36)	
pT			
pT1 + 2	79	36 (46)	
pT3 + 4	42	13 (31)	0.11
pN			
pN0	73	31 (42)	0.58
pN+	48	18 (37)	
Clinical stage			
I + II	50	23 (46)	
III + IV	71	26 (37)	0.30
G status			
G1	77	31 (40)	
G2 + 3	44	18 (41)	0.94
Tumor location			
Tongue	51	18 (35)	
Other sites	70	31 (44)	0.32
Tumor location			
Floor of the mouth	35	17 (49)	
Other sites	86	32 (37)	0.24
Tumor recurrence			
No	68	30 (44)	0.35
Yes	53	19 (36)	
Second primary carcinoma			
No	102	41 (40)	0.87
Yes	19	8 (42)	
Clinical status at the end of the follow-up			
Live and without recurrence	50	24 (48)	
Dead of index cancer	52	17 (33)	0.28
Censored	19	8 (42)	

Supplementary Table S4. Univariate Kaplan-Meier and Cox analysis to assess the association of clinicopathological variables on disease-specific survival in 125 OSCC patients.

Parameter	No Cases	Censored patients (%)	Mean survival time (95% CI)	HR (95% CI)	*P*
Tumor location					
Tongue	51	28 (55)	124.43 (94.00 – 154.86)	Reference	0.31
Other	74	44 (59)	120.47 (101.48 – 139.47)	0.75 (0.43 – 1.30)	
Tumor location					
FOM	37	23 (62)	109.11 (85.88 – 132.33)	Reference	0.56
Other	88	49 (56)	131.16 (108.62 – 153.70)	1.19 (0.64 – 2.21)	
Grade					
Well	80	44 (55)	127.85 (103.73 – 151.98)	Reference	0.59
Moderate-poor	45	28 (62)	121.63 (96.25 – 147.01)	0.85 (0.48 – 1.52)	
pT classification					
T1 + T2	81	53 (65)	151.82 (129.03 – 174.62)	Reference	0.001
T3 + T4	44	19 (43)	77.62 (54.19 – 101.04)	2.49 (1.44 – 4.30)	
pN classification					
N0	76	49 (64)	127.96 (109.43 – 146.49)	Reference	0.01
N+	49	23 (47)	108.58 (77.88 – 139.28)	1.92 (1.12 – 3.31)	
Stage					
I + II	52	36 (69)	140.09 (120.15 – 160.04)	Reference	0.002
III + IV	73	36 (49)	113.33 (87.73 – 138.93)	2.40 (1.33 – 4.32)	
SOX2 >10% nuclei					
Negative	72	37 (51)	116.37 (90.39 – 142.35)	Reference	0.07
Positive	49	32 (65)	146.66 (117.98 – 175.35)	0.59 (0.33 – 1.05)	

FOM: floor of the mouth.
